# Graphene-Based Biosensors for Molecular Chronic Inflammatory Disease Biomarker Detection

**DOI:** 10.3390/bios12040244

**Published:** 2022-04-14

**Authors:** Isidro Badillo-Ramírez, Yojana J. P. Carreón, Claudia Rodríguez-Almazán, Claudia M. Medina-Durán, Selene R. Islas, José M. Saniger

**Affiliations:** 1 Center for Intelligent Drug Delivery and Sensing Using Microcontainers and Nanomechanics (IDUN), Department of Health Technology, Technical University of Denmark, 2800 Kongens Lyngby, Denmark; 2 Instituto de Ciencias Aplicadas y Tecnología, Universidad Nacional Autónoma de México, Av. Universidad 3000, Coyoacan, Mexico City 04510, Mexico; yolanda.carreon@unach.mx (Y.J.P.C.); claudiar@comunidad.unam.mx (C.R.-A.); 314138921@quimica.unam.mx (C.M.M.-D.); selene.islas@icat.unam.mx (S.R.I.); 3 Instituto de Biotecnología, Universidad Nacional Autónoma de México, Avenida Universidad 2001, Cuernavaca 62210, Mexico

**Keywords:** biomarkers, graphene, biosensors, analytical platforms, inflammatory diseases

## Abstract

Chronic inflammatory diseases, such as cancer, diabetes mellitus, stroke, ischemic heart diseases, neurodegenerative conditions, and COVID-19 have had a high number of deaths worldwide in recent years. The accurate detection of the biomarkers for chronic inflammatory diseases can significantly improve diagnosis, as well as therapy and clinical care in patients. Graphene derivative materials (GDMs), such as pristine graphene (G), graphene oxide (GO), and reduced graphene oxide (rGO), have shown tremendous benefits for biosensing and in the development of novel biosensor devices. GDMs exhibit excellent chemical, electrical and mechanical properties, good biocompatibility, and the facility of surface modification for biomolecular recognition, opening new opportunities for simple, accurate, and sensitive detection of biomarkers. This review shows the recent advances, properties, and potentialities of GDMs for developing robust biosensors. We show the main electrochemical and optical-sensing methods based on GDMs, as well as their design and manufacture in order to integrate them into robust, wearable, remote, and smart biosensors devices. We also describe the current application of such methods and technologies for the biosensing of chronic disease biomarkers. We also describe the current application of such methods and technologies for the biosensing of chronic disease biomarkers with improved sensitivity, reaching limits of detection from the nano to atto range concentration.

## 1. Introduction

Inflammatory diseases are characterized by a complex range of disorders and conditions, comprising molecular and structural events in immune cell metabolism to promote tissue repair and recovery. The origin of inflammatory diseases can be due to infections by microorganisms, disorders in the immune system, blood clots, neurological conditions, exposure to various chemical substances and toxins, and others [1,2]. Inflammation is helpful to destroy or localize the injurious agent by inducing a sequential cascade of various leukocytes and biomarkers events, followed by physiological changes in the immune response to repair the damaged tissue or restore tissue homeostasis [3,4]; however, if acute inflammation persists for a long time, it becomes chronic inflammation, which includes the concomitant pump chemical messengers (biomarkers) and white blood cells [2,5].

The World Health Organization (WHO) considers diseases associated with chronic inflammation of great importance due to their increase and high costs of treatment, such as cancer, diabetes mellitus, cardiovascular diseases, allergies, oral diseases, obesity, stroke, arthritis, chronic obstructive pulmonary disease (COPD), COVID-19, and neurodegenerative disorders [6,7].

The increasing number of chronic inflammatory diseases has led to advanced efficient methods for the early detection of biomarkers, with the aim to support efficient diagnosis and therapy [8]. According to WHO, a biosensor is “any substance, structure, or process that can be measured in the body or its products and influence or predict the incidence of outcome or disease”. Detection of biomarkers, which express at low concentration, is a determining factor in chronic inflammatory diseases to understand the biomolecular process of inflammation, diagnosis, prognosis, and selecting an adequate therapy. Typically, chronic inflammatory disease biomarkers are performed by conventional methods, such as enzyme-linked immunosorbent assay (ELISA) [9], high-performance liquid chromatography (HPLC) [10], polymerase chain reaction (PCR) [11], DNA sequencing technology [12], medical imaging [13], or engineered probiotic microorganisms [14]. These methods can provide very-high sensitivity and specificity, but they involve expensive and bulky instruments and complicated time-consuming operations, which limit their implementation in clinics and for practical use to the patient; therefore, it is highly desirable to develop new POC technologies for identification [1,2], with the ability to be sensitive, simple, cost effective, portable, and easy to use, which could detect infectious diseases and monitor health conditions.

In recent years, biosensors technology has shown potential benefits in biomedicine as an alternative tool for detecting biomarkers of a disease at an early stage, or following the evolution of a biomarker in therapeutic responses in a quick, cheap, and straightforward way [15,16,17]. Together with new developments in nanotechnology, nanomaterials and bioelectronics have advanced the application of biosensors in clinical settings [18,19,20].

The IUPAC (International Union of Pure and Applied Chemistry) defines a biosensor as “a device that uses specific biochemical reactions mediated by isolated enzymes, immunosystems, tissues, organelles or whole cells to detect chemical compounds usually by electrical, thermal or optical signals”. Some review papers highlight the advancement in the nanotechnology of materials, allowing for the introduction of novel carbon-based materials, especially graphene, that have opened a new era in several fields of applications. Implementing graphene-based materials in biosensors for sensing and manufacturing has opened vast opportunities to develop simple, portable, and robust biosensors that might advance their future use in routine clinical applications.

Graphene and its derivatives, or graphene-derivative materials (GDMs), include pristine graphene, graphene oxide (GO), reduced graphene oxide (rGO), and graphene quantum dots are ideal materials for developing sensitive biosensors due to their versatility and properties, such as tunable electrical conductivity, wide surface area, and outstanding thermal conductivity [21]. Further, the large surface area of such materials allows them to be used for the surface immobilization of biomolecules, metallic particles, and label molecules to increase the sensing selectivity and sensitivity. Moreover, the efficient and low-cost production of these materials have advanced its use for diverse applications in biomedicine, catalysis, and environmental areas. In addition, the implementation of GDMs for bio-functionalization with different molecules or target biomolecules, such as aptamers, antibodies, DNA sequences, and enzymes, has shown a tremendous impact on robust biosensor development and its application to detect biomarkers of several diseases, such as diseases of the central nervous system [22,23], bacterial or viral infections [24,25], oral chronic inflammatory disease [26], cancer [27], diabetes [28], and biomarkers of inflammatory diseases [29,30].

This review presents the recent advances, properties, and benefits of GDMs for the biosensing of chronic disease biomarkers and their current applications for robust and sensitive detections through electrochemical, optical, portable, and miniaturized biosensor technologies. Further, in each section, we present the commonly used manufacturing technology of GDMs for fabricating simple, robust, portable, and miniaturized biosensors. Finally, we discuss the challenges, opportunities, and potential solutions of GDMs for the biosensing of biomarkers in chronic inflammatory diseases to advance their POC applications closer to the patient and in clinics.

## 2. Inflammatory Disease Biomarkers

Inflammation is initiated after a foreign particle, microorganism, chemical, or toxin stimuli enters the body, then the immune cells (dendritic cells, macrophages, histiocytes, Kupffer cells) [31,32,33] release diverse inflammatory mediators [34,35] and biomarkers to repair the damage, causing acute inflammation [36,37,38]. Acute inflammation is a defense process of the body against any aggression and is considered beneficial.

The inflammation begins with foreign particles’ contact with the pattern recognition receptors (PRRs) [39], nucleotide-binding oligomerization domain (NOD) [40], or neutrophil-lymphocyte ratio (NLR) [41]. NLRs detect pathogen-associated molecular patterns (PAMPs) or damage-associated molecular patterns (DAMPs) [42]. There are four classes of NLR: NLRP1, which recruits various caspases; recruitment domain-containing protein 4 (NLRC4) or protease-activating factor (IPAF), which activates the factor of proteases that act in the maturation of interleukins; (NLRP3) inflammasome sensor for immune control of diverse pathogens and inflammasome-forming receptor absent in melanoma 2 (AIM2) [43,44,45].

The border between acute and chronic inflammation is determined by duration. Acute inflammation is fewer than 48 h; if this period prolongs to weeks, months, or even years, it becomes a chronic inflammation, characterized by the presence of the inflammasome. The inflammasome [2,46] is a cytoplasmic multiprotein complex constituted by a nucleotide-binding oligomerization domain, leucine-rich repeat, and pyrin domain-containing (NALP1), apoptosis-associated speck-like protein containing a CARD (Picard/Asc), caspase-1, and caspase-5 [47]. The inflammasome induces the release of pro-inflammatory cytokines, called interleukins (ILs), which play a key role by regulating interactions between leukocytes; the main pro-inflammatory cytokines include IL-1β, IL-18, and IL-33 [48,49], and alarmins, such as IL-1α, IL-33, and IL-6 cytokines [50,51]. Finally, the process can trigger tissue destruction, fibrosis, and necrosis [52,53].

The presence of molecular biomarkers in chronic inflammation allows the understanding, prediction, diagnosis, and evaluation of responses to therapeutic interventions through clinical trials. Nevertheless, there are no specific biomarkers for each chronic disease due to the high complexity of molecular and cellular interactions generated during the inflammatory response [36,54,55]; however, the main goal in diagnosing inflammation is to detect potential biomarkers involved in chronic inflammation, such as cytokines, that take part in cell proliferation and differentiation. In addition, other inflammatory proteins, and enzymes such as superoxide dismutase (SOD) [56] and cyclooxygenase (COX)-2 [57], have an essential role in developing inflammation-related diseases. In addition, non-protein molecules that are the product of oxidative stress, such as 8-hydroxy-2-deoxyguanosine, are also potential biomarkers in inflammatory diseases [58,59]. The identification of new biomarkers of inflammatory diseases and the increase in their expression is mainly performed with proteomics, a technique that determines the amino acid sequence of a set of proteins present in a sample; however, this technique is expensive and does not yield real-time results [60,61]. For this reason, many researchers are focusing on developing sensitive and specific techniques that provide a diagnosis in real-time, such as biosensors. Table 1 lists the main molecular biomarkers on which the diagnosis of chronic inflammatory diseases has been focused.

## 3. Graphene and Graphene-Derivative Materials, Classification, and Properties

**Figure 1:**
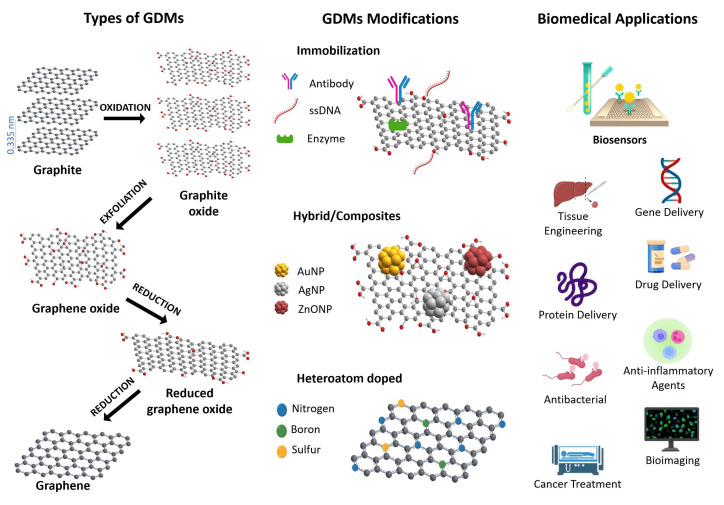
Schematic representation for the types of GDMs and route of graphite to graphene, its modifications, and potential applications in several research fields.

Graphene and its derivatives (GO, rGO, GQD, and doped graphene) have opened a new era in materials processing due to their unique physicochemical properties for the robust technological development of sensors and biosensors, with potential applications in several research fields (Figure 1). This section describes the characteristic properties of graphene and its derivatives.

### 3.1. Graphene

Carbon is the constitutive element of graphene and GDMs. The structure and properties of these materials are closely related with the versatility of the carbon chemical bonding; its capacity to promote one paired electron from the 2s to the 2p atomic orbitals allows it to hybridize their atomic orbitals in different ways. The $sp^{2}$ hybridization in carbon materials confers a planar 2D structure, where each carbon has three covalent-bonded neighboring carbon atoms. This planar topology, characteristic of a graphene single-layer, provides an exceptionally high surface area, thermal and electrical conductivity, high mechanical strength and stretchability, chemical inertness, and intrinsic biocompatibility [108,109]; however, from a chemical point of view, the reactivity of graphene layers is low and only π-π interactions or van der Waals forces should be expected in the presence of other molecular species or biological structures; therefore, the hydrophobicity of graphene restricts its possibilities of interaction with biomaterials and biomolecules. For these reasons, the use of the aforementioned GDMs is a versatile strategy to expand and modulate the possibilities of chemical interactions between graphene materials and biomolecules [109]. The hydrophilic character of GDM facilitates immobilization of the biostructure, which often leads to an improvement in the sensitivity of biosensors, as described in the additional section.

### 3.2. Graphene Oxide (GO)

GO is characterized by containing several oxygen functional groups, such as hydroxyl, carboxylic acid, epoxy, and esters, that provide reactive surface sites, good dispersibility and stability in aqueous media, and favorable binding sites for molecular functionalization and biomolecular immobilization [110,111]. GO is commonly obtained from graphite through the Hummers, Brodie, and Staudenmaier methods [112,113]. In these methods, the graphite undergoes a strong chemical oxidation process, allowing one to obtain graphite oxide, which is subsequently exfoliated to obtain highly dispersed hydrophilic GO layers (Figure 1).

After oxidation of GO, the graphite carbon network modifies substantially; the intercalation and attachment of oxygenated species into the initial graphite layered structure leads to significant structural changes. For example, when an oxygenated group binds to a C sp${}^{2}$ atom, it changes its hybridization to sp${}^{3}$ and the 6C aromatic planar ring changes to no planar 6C chair/boat ring. Because of these changes, the GO carbon network is composed of aromatic and non-planar aliphatic domains, resulting in the corrugated texture of the GO surface. In addition, due to the high oxidative chemical attack, point and extended defects are introduced into the GO layers, which enhances the surface electrical charge. Moreover, the presence of oxygenated polar species confers GO an improved dispersibility in aqueous media, allowing its applications in biological media conditions. Consequently, the chemical structure and properties of the former flat and aromatic C layer of GO are significantly changed, and its reactivity increases enormously.

GO is considered a non-stoichiometric, polydisperse, and amphiphilic compound. The C/O ratio defines its degree of oxidation, while its reactivity depends on the types and amount of the oxygenated species attached to the edges and the basal plane of the carbon network [114]. Nevertheless, GO preserves some distinctive physicochemical properties of graphene, such as large surface area, small size, amphiphilicity, and fluorescence quenching ability [115,116]. These versatile properties make GO an excellent material for technological development in biomedical and biosensor developments.

Additionally, nanocomposites of GO with metals, polymers, and similar nanomaterials have extended the various biotechnological applications in biosensing [117]. Interestingly, recent studies have shown the potential anti-inflammatory properties and biocompatibility of GO, depending on its size and concentration [118,119]; however, it is still necessary to understand the full effects of GO on the innate immune system. Further studies focusing on this field could potentiate the use of GO as an anti-inflammatory agent for clinical applications.

### 3.3. Reduced Graphene Oxide (rGO)

Reduced graphene oxide is the product obtained after the chemical, electrochemical, thermal, or solvothermal reduction in GO [120]. This reduction process removes significant amounts of the oxygenated species attached to GO. The type and quantity of oxygenated species removed depend on the experimental conditions, but in general terms, the oxygenated groups with less chemical stability remove preferentially; this is the case of the epoxy groups attached to the basal plane of GO (Figure 1). Other groups attached to the edges of GO flake, such as carboxylic or hydroxylic, are also removed but to a lesser extent. Other critical structural changes resulting from the elimination of oxygenated species are the decrease in the number of C atoms with sp${}^{3}$ hybridization and the consequent increase in sp${}^{2}$ hybridized C, leading to a partial recovery of the aromatic domain size network of pristine graphene. The derived effects of these structural changes are the lower dispersibility of rGO in aqueous media, due to the remotion of polar groups and its higher aromatic character. Additionally, the number of extended defects in the carbon network also increases due to the structural stress associated with the chemical reduction.

The possibility to have control of the structural modifications of rGO makes it a versatile material with modulable intermediate properties between pristine graphene and GO. The adequate modulation of the binomial structure/properties of rGO allows one to obtain a graphene derivative that contains polar and non-polar aromatic domains on its surface, providing the dispersibility [117] and electrical conductivity required to develop optimized detection devices for a specific analyte [121]. The possibility of modulating the properties of rGO is beneficial for the design of biosensors with improved specificity for specific biomolecules through the promotion of desired molecule/substrate interactions.

### 3.4. Graphene and GDMs Modifications

Chemical functionalization and doping of graphene (surface modifications) are the main methods to manipulate and modulate its physical and chemical properties [122,123,124]. The surface functionalization of graphene can be classified into covalent and non-covalent [123]. Covalent functionalization involves the attachment of other functional groups through chemical attack on the π bonds of unsaturated carbons [125], resulting in substantial modifications of the electronic and geometric properties of graphene [123]. Indeed, covalent functionalization results in the disappearance of graphene’s unique structure and properties, leading to the formation of a graphene-derived material.

On the other hand, the non-covalent functionalization occurs when graphene interacts with other chemical functional groups via van der Waals, π–π, ionic, or hydrogen bonding forces. Typical examples are the interaction with aromatic molecules (π–π stacking), hydrogen bonding between carboxylic groups at the edges of GDMs and biomolecules, and ionic interaction of amino protonated groups and carboxylates. This type of functionalization is frequently used when the stable dispersion of graphene or GDMs in different media are needed, or for the attachment of molecules acting as bridges between GDMs and specific biomolecules, as is the case with the fabrication of highly selective biosensors for a family of biomolecules. Non-covalent functionalization typically preserves some of the properties of initial graphene or GDMs, while the bonding between the wrapping molecules is weaker than the covalent functionalization [122]. Following this methodology, many functionalization reactions [126] have been reported in the literature to expand the catalog of GO and rGO interactions to biostructures. This approach makes it possible to obtain functionalized graphene oxides with many chemical affinities towards different biostructures.

Moreover, the introduction of heteroatoms as dopants into the 2D structural arrangement of pristine graphene has shown interesting properties to develop new materials for diverse applications (Figure 1). For example, nitrogen doping graphene can enhance electron transfer, improve graphene catalytic activity and binding ability, and at the same time, can increase the biocompatibility and sensitivity of graphene-based biosensors.

## 4. Biosensors Techniques Based on GDMs, Fabrication, and Applications in ID

Biosensors are classified according to their type of biorecognition (e.g., antibody, aptamer, enzyme) or due to the type of transducing element, which most commonly includes electrochemical, optical, and mass-based biosensors; however, in the field of biosensors developed with GDMs, the electrochemical and optical methods are preferred for the following reasons: (i) the excellent GDMs electronics and optical properties that significantly improve the sensing type of recognition and (ii) their versatility for manufacturing and material processing that, when coupled with other appropriate materials, allow the novel fabrication of robust biosensor devices.

The fabrication of GDM-assisted biosensors implies diverse methodological approaches, depending on he type of recognition; e.g., optical or electrochemical. The most-frequent GDMs used for this purpose are flat graphene sheets, micrometric GO or rGO flakes, and nanosheets of GO or hybrid materials. A description of the design and fabrication of biosensors with GDMs is shown in the following sections, according to their type of recognition. GDMs has a strategic role in the sensing element by enhancing electro-optical recognition effects and, at the same time, facilitating the anchoring of target analytes to be specifically recognized. In this section, we present the main types of electrochemical and optical biosensors based on GDMs, the bases of their design and fabrication and the strategies to develop miniaturized, remote, and wearable biosensor devices. Finally, each section describes their novel applications to detect inflammatory disease biomarkers.

### 4.1. Electrochemical Biosensors

Electrochemical biosensors based on GDMs are the most employed devices for the biosensing of biomarkers of inflammatory diseases. These types of biosensors provide practical advantages, such as their simple design and low-cost fabrication, minimal power requirements, friendly user interfaces, ease of miniaturization, robustness in the measurement, and the requirement of small sample volumes.

**Figure 2:**
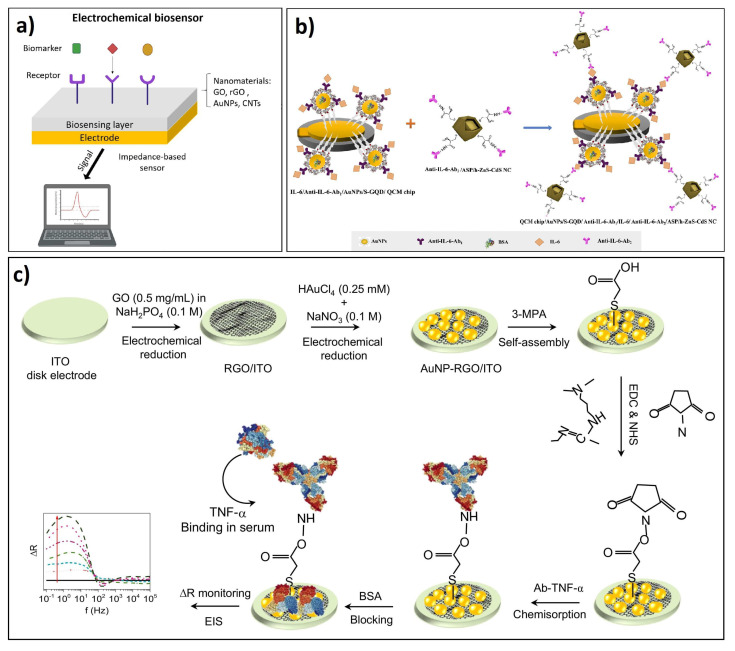
Electrochemical biosensors. (**a**) Schematic illustration of the general design of an impedance-based biosensor. (**b**) Surface design of a sandwich-type electrode biosensor based on functionalized sulfur-doped GQD for IL-6 detection. (**c**) Design and manufacture of ITO disk electrode for biosensing of TNF-α. *Source*: (**b**,**c**) reproduced from [127,128] with permission of Elsevier.

An electrochemical biosensor is a self-contained integrated device that uses a bio-receptor (antibodies, proteins, nucleic acids, enzymes) as the biological recognition element. The general components of an electrochemical biosensor are shown in Figure 2a. After selectively reacting with the target analyte, an electrical signal is produced that is finally detected through a suitable sensing device. Electrochemical biosensors can be classified according to the type of transduction mode that is used for measuring the analytical response, and according to the type of electrochemical reaction that takes place between the electrodes or at the surface of the electrode. In this sense, they are classified as: (1) amperometric, if a measurable current signal is involved; (2) potentiometric, when a potential or accumulated charge is measured, and (3) conductimetric, if there is a change in the conductivity of the medium of reaction.

In the following paragraphs, we describe the sensing detection, their design and requirements assisted with GDMs for biosensing purposes.

#### 4.1.1. (A) Amperometric

Amperometry sensors measure a change in the current obtained after applying a fixed potential between a working and a reference electrode in an electrolytic reaction. The current is measured continuously in an amperometry transducer after an electrochemical reaction of reduction or oxidation occurs at the working electrode, and this response is proportional to the concentration of the analyte. The concentration of the target molecule is determined by measuring the value of a peak current according to any selected potential. In amperometry, the change in current is monitored as a function of time and at constant potential; however, if the potential is scanned along a potential range, it is called the voltammetry-sensing method. Standard voltammetry methods include cyclic voltammetry (CV), differential pulse voltammetry (DPV), square-wave voltammetry (SWV), and polarography. The advantages of these methods rely on their superior sensitivity and wide measurement dynamic range. Amperometry detection is preferred for bioanalytical sensor devices due to its simplicity and very-low limit for analytic detection and quantification.

Electrodes are key components of an electrochemical cell, which is composed by: (i) a working electrode, usually a solid conductive material, such as carbon, gold, or platinum; (ii) a reference electrode, typically an Ag/AgCl system; (iii) a platinum auxiliary electrode. These electrodes require high stability and performance and, ideally, miniaturized dimensions.

GDM-based electrodes may present variations in fabrication processes according to the type of graphene material used, pristine graphene, graphene derivatives, CVD-grown graphene, or chemically modified graphene. In all these cases, recently developed manufacturing methods, such as screen-printed electrodes (SPEs), are highly suitable for their production.

#### 4.1.2. (B) Potentiometric

Potentiometric methods consist of transducers, measuring the potential change of a reference electrode against the working electrode, which usually contains the bioreceptor agent in an electrochemical cell under negligible current conditions. The measured potential can determine the concentration of an analyte in the solution, such as ions. Typical potentiometric electrodes include the glass pH or ion-selective electrodes. Further, potentiometric electrodes can work as biosensors by incorporating a biological agent on their surface, such as an antibody aptamer. After the reaction with a target analyte, an ion or charged species is obtained and detected in the second electrode. The primary type of potentiometric sensors includes transducers based on polymer or membrane ion-selective electrodes (ISE), solid electrodes, screen-printed electrodes, solid-state electrodes, and ion-selective field-effect transistors (ISFET). This types of biosensor offer practical advantages such as the requirement of small sample volumes, reduced or absent chemical cross talk in the sample measurement.

The FET effect is commonly used in the working principle for designing flexible graphene electrochemical biosensors. The design of an FET biosensor consists of a source (S) and a drain (D), placed on a solid substrate such as SiO${}_{2}$/Si, and a gate (G) between D and S. In the gate region, monolayers of graphene materials are placed to immobilize biological substrates and to enhance the electron transfer efficiency by increasing the electron carrier concentration after a target molecule causes a change in the charge distribution. In a complete FET device, the change in conductivity is measured after applying an external field. The sensitive electrical response in an FET sensor is highly dependent, besides the electrical properties, on the length and uniformity of the surface material; thus, GDM has been extensively used as substrates for FET fabrication in the so-called graphene-based field-effect transistor (GFET) [129]. The FET/GFET principle has been used principally to design compact, remote, and wearable biosensors for detecting relevant inflammatory biomarkers, as is described in the following section.

#### 4.1.3. (C) Conductimetric

Impedance is the most-used technique in conductometric biosensors. Electrochemical impedance spectroscopy (EIS) works by measuring the resistive and capacitive responses of an electrochemical cell after a perturbation with a small AC excitation signal. An impedance spectrum is obtained by measuring the current through the electrochemical cell after the detection event. This technique is frequently used for affinity biosensors, such as immunosensors, to monitor antibody–antigen binding on an electrode surface.

Usually, in an impedance biosensor, the surface of the electrode can be immobilized with a particular biological recognition element, such as antibodies. The recognition of the analyte with the target element, causes a change in the electron resistance between the biofunctionalized electrode and the solution. The advantage of EIS is the ability to measure electron transfer at a high frequency and mass transfer at a low frequency, which allows one to follow a binding conjugation event in real-time in a label-free mode, with additional advantages such as high sensitivity, fast assay, low assay cost, ease of detection, and short time of detection response.

#### 4.1.4. (D) Electrochemiluminescence

Electrochemiluminescence (ECL) is characterized by the conversion of electrical energy into light-emitting radiative energy. The process involves the formation of intermediate reactive species at the surface of an electrode after applying a voltage, such species undergo electron-transfer reactions with a consequent emission of light. Conventionally, Ru and their complexes are preferred materials for ECL reagents; however, GO/rGO composites and GQDs have been implemented for developing ECL aptasensors, due to their chemical stability and surface area modifications, which have been applied for detection of several biomolecules [130]. In addition, these biosensors provide high sensitivity, suppressed background scattered light, multiple species detection, and simple instrumentation requirements.

### 4.2. Design and Fabrication of Electrochemical Biosensors Based on GDMs

In general, the fabrication of platforms for electrochemical biosensors requires the design and adequate electrode fabrication, mainly focused on surface modifications of electrodes that use the GDMs as the sensing part, which is typically functionalized and immobilized with specific target biomolecules. For example, in conventional antibody electrochemical immunosensors, the surfaces of GO single layers are immobilized with high selective antibodies, providing very specificity recognition. During the functionalization surface, it is necessary to form amine binding with antibodies through intermediate compounds, such as 1-Ethyl-3-(3-dimethyl aminopropyl) carbodiimide (EDC), activating carboxylic groups. In some cases, the design of labeled electrochemical biosensors implies incorporating labeled aptamers or antibodies with fluorescent or redox probes allowing specificity after the electrochemical recognition with the biomarker. Following this principle, Cao and coworkers fabricated an amperometric biosensor by modifying the surface of a glassy carbon electrode with a single layer of GO, acting as a signal amplifier, where hairpin aptamers, with specific recognition to interferon-γ, were loaded with Ru redox probes, acting as biorecognition moieties through the electrochemical signal. This device showed biocompatible properties for an in vivo measurement of IFN-γ over 48 h in enteritis mice [131].

Furthermore, the sandwich-type design method is a fabrication strategy highly preferred to prepare selective electrochemical immunosensors; in this case, GDMs are placed on the electrode surface, where antibodies are covalently bonded to confer specificity sensing recognition of inflammatory cytokines. For example, Qi and colleagues designed a nano sandwich device based on GO as the electron transfer and the signal reporter. The sandwich device consisted of modifying a gold electrode with a single layer of GO, with the surface modified through a capture monoclonal anti-IL-6 (Ab1); then, a second GO layer was labeled with Nile blue (NB) and immobilized IL-6 antibody (Ab2). As a result, the cytokine IL-6 detection was performed through the electrochemical signal of NB after recognition [132]. In addition, similar designs have incorporated metallic nanoparticles, mainly Au or Ag, functionalized with antibodies, to provide specificity, and enhance the electrochemical detection of cytokines [133].

Furthermore, the fabrication of GDMs electrodes has been recently improved by jet-printed methods, such as finger-widths printed from graphene-nitrocellulose ink, where antibodies can be further covalently immobilized over the electrodes for sensing recognition of inflammatory cytokines [134]. Moreover, manufacturing micro disk electrodes with rGO and AuNPs on an indium oxide (ITO) substrate have allowed us to obtain robust antibody-functionalized electrodes to enhance the electrochemical specific recognition of IFN-γ [127] (see Scheme in Figure 2b). Further, additional strategies to improve the electrode performance for sensitive detection of IL-6 was achieved by coating GO electrodes with metals or metallic NPs (mainly Au or Ag) and thin films that contain rGO and polymer formation materials such as polyethylenimine or Nafion [132,135].

#### Applications of Electrochemical Biosensors in Detection of ID Biomarkers

Although most of the electrochemical techniques are versatile, amperometric, voltammetric, and EIS have been highly preferred for biodetection [136,137,138]. Electrochemical sensors enhanced through the use of GDMs have found numerous and promising applications in the literature for the sensitive detection of inflammatory biomarkers, especially for inflammatory cytokines. Interleukins are preferred targets for single or multiple detections with electrochemical approaches.

IL-6 has been the most employed cytokine biomarker for detection with electrochemical methods, such as impedance [139,140], CV [128], DPV [135], EIS [128], FET [141,142,143], and SWV [132,144]. Several sensing platforms have been designed by integrating the GDM within the sensor recognition part, such as electrodes, typically decorated with aptamers or antibodies against IL-6, aiming to provide recognition specificity. Moreover, these developments have been assessed for specific detection in diverse types of samples, from buffer solution to clinical serum, showing fundamental capabilities in specificity and high sensitivity. For example, Atar and Yola [128] have recently shown one of the most-sensitive electrochemical designs for IL-6 detection in prepared serum samples through a quartz crystal microbalance (QCM) immunoassay method. The design consisted of fabricating a sandwich-type electrode of Au NPs functionalized sulfur-doped graphene quantum dot (AuNPs/S-GQD) and hollow ZnS–CdS nanocage (h-ZnS-CdS NC), both immobilized with anti-IL-6 for selective detection of IL6 analyte through CV and EIS measurements, leading a LOD of 3.33 fg/mL with high selectivity and stability in the presence of interference protein biomarkers including BSA, VEGF, p53, IL-8, and IL-1β (see Scheme in Figure 2b).

On the other hand, the simultaneous detection of relevant inflammatory cytokines with novel graphene materials has been applied to detect IL-6 altogether with IL-17 [145], TNF-α, IFN-γ, and IL-1β [146,147]. In those designs, the presented approach was based on sandwich immunoassays, consisting of two specific antibodies, one on the surface and the other in the signal reporter, which allowed identifying the biomarker through separate electrochemical outputs with SWV and EIS methods. For example, Wei and colleagues developed a sandwich GO immunosensor loaded with Nile blue, methyl blue, and ferrocene redox probes, which conferred specificity against IL-6, IL-1β, and TNF-α, respectively; their detection was performed through SWV, according to the change in the electrochemical signal from signal reporters, reaching a LOD of 5 pg/mL for the three cytokines in spiked whole mouse serum, and non-interference was observed from BSA, IgG, PSA, or CA-125 proteins [146].

IFN-γ and TNF-α are key biomarkers of high relevance in inflammatory diseases, which have been detected using GDMs and through amperometry [131,133,148], impedance [127,134], EIS [147], and voltammetry [149] methods. For example, Yagati and colleagues developed ITO micro disk electrodes modified with rGO and Au NPs immobilized antibodies (see design in Figure 2c). EIS measurements were performed by detecting the resistance change after recognition of TNF-α reaching a LOD of 0.67 pg/mL and 0.78 pg/mL, in PBS solution and human serum, respectively, without interference of common serum proteins such as BSA or CRP (Figure 2c) [127]. On the other hand, Cao and coworkers developed a biocompatible and recyclable device based on GO and structure-switching aptamers, where GO immobilized hairpin specific aptamers were loaded with redox probes for the biorecognition of IFN-γ, allowing its in vivo quantification after secreted by cultured immune cells at a LOD down to 1.3 pg/mL. This device was further implanted into subcutaneous tissue mice, showing continuous measurement of IFN-γ over 48 h and multiple cycles of regeneration, without the interference of nonspecific proteins, such as IgG, TNF-α, BSA, or IL-6 [131].

Furthermore, related methodologies based on GDMs, and electrochemical biosensors were developed to detect additional critical inflammatory interleukins, such as IL-4 [131], IL-8 [150,151,152], IL-10 [134], IL-13 [153], IL-15 [154], and IL-22 [155]. In these works, GDMs and their nanocomposites played a key role to improve the detection of cytokines human biological fluids, such as saliva and serum, cell lysates, and tissue extracts. For example, Verna and colleagues developed an immunosensing platform for noninvasive onsite detection of IL-8 based on ZnO-rGO supported on an ITO substrate, immobilized with anti-IL-8, allowing a LOD of 51.53 pg/mL with sensitivity detection of 2.46 μA mL/ng in saliva samples [152].

On the other hand, several reports have shown the detection of additional relevant protein biomarkers related to inflammatory diseases, such as cTnI, as a critical biomarker of acute myocardial infarction detected by CV [156,157], EIS [158,159,160,161], DPV [162,163,164], amperometry [165], and ECL [166,167,168]. In these examples, the reached LOD was lower than 1 pg/mL in clinical serum samples. Furthermore, in the last years, a significant improvement for cTnI detection was achieved by Gou and colleagues by synthesizing a functionalized GQD to be integrate into a label-free ratiomeric ECL immunosensor for the sensitivity detection of cTnI, which allowed researchers to reach LOD of 0.35 fg/mL in buffer solution and around 3.2 pg/mL in clinical human serum samples; selectivity of the immunosensor was evaluated with relevant interfering proteins such as Mb, hFABP, BSA, HAS, and IgG [168].

BNP is a crucial biomarker for heart failure, which has been detected through FET approaches, assisted with B/N codoped GO gel material to develop a FEB system, leading to detect BNP at a lowest LOD of 100 fM [169]. Similarly, BNP detection was improved by incorporating PtNPs-decorated r-GO in an FET biosensor, leading to achieve a LOD of 100 fM, and its performance was assessed in post filtered human whole blood samples [170].

CRP is another key inflammatory biomarker that has been detected by developing graphenic substrates, such as rGO-SPCE, conductive nano-hybrid material of Au NPs/IL-MoS2 onto GO and electrodeposited-sized GQDs over a SPCE surface, and detection of CRP was performed through EIS, CV, amperometry, and chronoamperometry, leading to reach a lower LOD of 3.3 pg/mL, in the best condition, in PBS and in spiked human serum [171,172,173,174].

In addition, a sandwich-type electrode based on GO/AuNPs, anti-total PSA, and anti-free PSA antibodies was fabricated for the total and free detection of PSA antigen, in the order of 0.2 ng/mL and 0.07 ng/mL, respectively, by performing CV and SWV measurements [175]. Further, selectivity was evaluated against related tumor markers such as BHCG, Alb, CEA, CA125, and CA19-9.

On the other hand, MDA, a relevant biomarker in oxidative stress conditions, has been detected with a label-free approach by preparing polyarginine-GQD, which showed excellent electroactivity towards MDA, allowing its direct detection in exhalated condensated breath (EBC) samples, reaching a LOD of 0.329 nM [176].

Furthermore, 8-OHdG is another important inflammatory biomarker of high relevance for oxidative stress, diabetic nephropathy, cancer, and neurodegenerative diseases that has also been sensitively identifies mainly with label free approaches, by detection the oxidation signals of 8-OHdG with the development of several sensing platforms and electrodes, that combined several GDMs composites and electrochemical methods, including CV [177,178,179,180], DPV [181,182,183,184,185], amperometry; SWV [186], SWV [187]; EIS [188]. The performance of such developments allowed the 8-OHdG detection in complex samples, such as clinic urine, without the interference of co-existant molecules in urine, such as uric acid.

A detailed description of relevant biomarkers for inflammatory diseases and their detection assisted with GDMs electrochemical biosensors is presented in Table 2.

### 4.3. Optical Detection Biosensors

In an optical biosensor, the recognition of a biological event or a biomarker is performed through a detectable optical signal by means of a transducing unit. These types of sensors provide high sensitivity, fast detection, low noise, high signal stability, and very low external disturbance. These features have extended the field of applications in biomedical studies, clinical diagnosis, and environmental monitoring [189]. Moreover, the capabilities optical biosensor can be boosted by combining the unique properties of GDMs; however, their applications for the inflammatory diseases biomarkers detection assisted with GDMs are still under development with a significantly lower number of reports in the literature, in comparison with electrochemical sensors. Optical biosensors based on GDMs can be divided into surface plasmon resonance (SPR), graphene-enhanced Raman scattering (GERS), fluorescence, and colorimetric methods. In this section, different reported examples of graphene-based optical biosensors for inflammatory biomarkers detection are discussed and their main features are compared and summarized in Table 3.

**Table 2:** Summary of electrochemical biosensors assisted with GDMs for detection of inflammatory disease biomarkers.

Biomarker	Sensing Platform Design	Surface Modification	Detection Method	LOD	Detection Range	Type of Sample	Ref.
IL-6	ErGO-AuPdNPs and AgNPs	anti-IL6	DPV	0.059 pg/mL	0.1–100,000 pg/mL	PBS buffer and serum proteins	[135]
	GFET-based BioMEMS platform	PTDA aptamers	Impedance	8 pM	100 pM–100 nM	PBS buffer	[139]
	Ab2-GO-NB/Au-ph-GO-PPC	anti-IL-6-NB label	SWV	1 pg/mL	1–300 pg/mL	mouse cells and live mice	[132]
	AuNP-graphene-silica/ITO	HRP-Ab2-AuNP-PDA@CNT label	Impedance	0.3 pg/mL	1–40 pg/mL	PBS buffer and clinical serum	[140]
	electrolyte-gated GFET	Aptamer	GFET	0.139 pM	0.0015–100 nM	PBS buffer	[141]
	GNRs-modified HSPCE	anti-IL-6/PS @ PDA/Ag NPs	SWV	0.1 pg/mL	1 × 10${}^{-5}$–1 × 10${}^{-3}$ ng/mL	PBS buffer and clinical serum	[144]
	crumpled graphene FET	Antibody anti-IL-6	FET	1 aM		PBS buffer solution	[143]
	AuNPs/S-GQD/QCM chip and h-ZnS-CdS NC	anti-IL-6	CV and EIS	3.33 fg/mL	0.01–12.0 pg/mL	Buffer solution and spiked plasma	[128]
	ECVDGO-liquid-gated FET	anti-IL-6	FET	1.53 pg/mL	1.53–300 pg/mL	PBS buffer	[142]
Il-6 and IL-17	GC/graphene-AuNps-Ab1	AuNP-anti-IL-6 and Ps-anti-IL-17	SWV	IL-6: 0.5 pg/mL; IL-17:1 pg/mL	IL-6: 1 pg/mL–1 ng/mL; IL-17: 2 pg/mL–1 ng/mL	PBS solution and clinical serum	[145]
TNF-α	Au-RGO-ph-AuNP-ph-PPC	anti-TNF-α	Amperometry	0.1 pg/mL	0.1–150 pg/mL	PBS buffer and live BV-2 cells	[133]
	AuNP-rGO/ITO microelectrode	anti-TNF-α	EIS	0.43 pg/mL	1–1000 pg/mL	PBS buffer	[190]
	AuNP-RGO/MDEAs	anti-TNF-α	EIS	0.78 pg/mL	1–1000 pg/mL	Buffer and spiked human serum	[127]
	Ag@Pt-rGO nanocomposite	TNF-α aptamer	DPV, SWV	2.07 pg/mL	2.07–60 pg/mL	PBS buffer and spiked human serum	[149]
TNF-α, IL-6, and IL-1β	Ab-GO-loaded NB, MB, Fc	anti-IL-6, anti-IL-1β, and anti-TNF-α	SWV	TNF-α: 5 pg/mL; IL-6: 5 pg/mL IL-1β: 5 pg/mL	TNF-α : 5–200 pg/mL; IL-6: 5–150 pg/mL; IL-1β: 5–200 pg/mL	PBS buffer and spiked whole mouse serum	[146]
TNF-α and IFN-γ	Au-GO/SA	biotin-IFN-γ aptamer-MB; biotin-TNF-α aptamer-Fc	CV, SWV and chronoamperometry	5 pg/mL for each cytokine	IFN-γ: 2–300 pg/mL; TNF-α: 5–200 pg/mL	Tris buffer and spiked serum and artificial sweat	[148]
IL-6, TNF-α and IFN-γ	monolayer graphene/Cu electrode	label-free	EIS	175 kDa total cytokine mass		Clinical patient serum	[147]
IFN-γ	GC-ph-GO	Aptamer(Ru), label	CV and SWV	1.3 pg/mL	1.3–210 pg/mL	Tris buffer, peripheral blood cells and mice interstitial fluids	[131]
IFN-γ and IL-10	AJP graphene IDE	anti-IFN-γ and anti-IL-10	EIS	IFN-γ: 25 pg/mL and IL-10: 46 pg/mL	IFN-γ: 0.1–5 ng/mL; IL-10: 0.1–2 ng/mL	PBS buffer and bovine implant serum	[134]
IL-8	Fe${}_{3}$O${}_{4}$@GO@MIP nanoparticles	MIP NPs	CV and DPV	0.04 pM	0.1 to 10 pM	phosphate buffer and human saliva	[150]
	anti-IL8/AuNPs-rGO/ITO	anti-IL-8	DPV	72.73 pg/mL	500 fg/mL to 4 ng/mL	PBS buffer and spiked human saliva	[151]
	anti-IL8/ZnO-rGO/ITO	anti-IL-8	DPV	51.53 pg/mL	100 fg/mL–5 ng/mL	PBS buffer and spiked human saliva	[152]
IL-4	rGO/chitosan/GCE	anti-IL-4	EIS	80 pg/mL	0.1 to 50 ng/mL	Phosphate buffer	[191]
IL-13	MWCNTs/GQDs nanocomposite	BCAb and hybrid MWCNTs/GQDs-HRP-DAb	CV and EIS	0.8 ng/mL	2.7 to 100 ng/mL	PBS buffer, cells lysates and tissues extracts	[153]
IL-15	GO/SPCE electrode	anti-IL-15	DPV	3.51 ng/mL	5–100 ng/mL	PBS buffer	[154]
IL-22	PDDA-G/AuNPs/ITO	anti-IL-22	DPV	0.5 pg/mL	5 to 5000 pg/mL	PBS buffer	[155]
cTnI	APTES/nMo${}_{3}$Se${}_{4}$-rGO/ITO	anti-cTnI	CV	1 fg/mL	1 fg/mL–100 ng/mL	PBS buffer	[156]
	PrGO/GC	anti-cTnI	EIS	0.07 ng/mL	0.1–10 ng/mL	PBS buffer and clinical samples	[158]
	AgNP/MoSO${}_{2}$/rGO	Aptamer anti-cTnI	EIS and DPV	0.27 pg/mL	0.3 pg/mL to 0.2 ng/mL	Tris-HCl buffer	[159]
	LSG-ZnFe${}_{2}$O${}_{4}$ aptasensor	Aptamer anti-cTnI	CV and SWV	0.001 ng/mL	0.001 ng/mL to 200 ng/mL	Buffer and spiked in human serum samples	[157]
	GQDs/AuNPs/SPGE	anti-cTnI	SWV, CV and EIS	0.1 pg/mL buffer; 0.5 pg/mL serum	1–1000 pg/mL buffer; 10–1000 pg/mL serum	Sodium acetate buffer and spiked human serum	[160]
	N-doped porous-rGO	Tro4 aptamer	DPV	1 pg/mL	0.001–100 ng/mL	PBS buffer and spiked human serum and saliva	[162]
	Microfluidic APTES-Mn${}_{3}$O${}_{4}$-RGO/ITO	anti-cTnI	EIS	8 pg/mL	0.008–20 ng/mL	PBS, spiked and clinical serum samples	[161]
	GOPRu–Au hybrid	anti-cTnI	ECL-RET	3.94 fg/mL	10 fg/mL–10 ng/mL	PBS and spiked human serum	[166]
	SPE-rGO/PEI	anti-cTnI	DPV	1 pg/mL	1 pg/mL–10 ng/mL	PBS buffer and clinical serum samples	[164]
	Au NCs-GQDs-Ab2	anti-cTnI	ECL	354.2 fg/mL	500 fg/mL–20 ng/mL	PBS and spiked human serum	[167]
	Apt-CES-GO/SPE	anti-cTnI	Amperometry	0.6 pg/mL	1.0 pg/mL to 1.0 μg/mL	Sodium phosphate buffer	[165]
	CuNWs/MoS${}_{2}$/rGO	aptamer AcTnI	DPV	0.1 pg/mL	0.5 pg/mL–100 pg/mL	PBS buffer and spiked human serum	[163]
	ABEI@GQDs	anti-cTnI	ECL	0.35 fg/mL	1.0 fg/mL to 5.0 pg/mL	Buffer and clinical serum samples	[168]
BNP	BN-GO	BNP Ab (50E1)	FET	10 aM	10 aM${}^{-1}$ μM	Buffer solution	[169]
	PtNPs-rGO	anti-BNP	FET	100 fM	100 fM${}^{-1}$ nM	PBS buffer and whole human blood	[170]
CRP	rGOx-GA-BSA/Au	anti-CRP	CV	0.492 μg/mL	0.63–3.76 μg/mL	Buffer, serum and diluted whole blood	[173]
	Ir NPs/GO-DN	anti-CRP	Chronoampe- rometry	3.3 pg/mL	0.01–100 ng/mL	PBS buffer and spiked in human serum	[172]
	SPCE/GQDs/anti-CRP	anti-CRP	Amperometry, DPV	0.036 ng/mL	0.5–10 ng/mL	PBS and spiked in Ringer lactate solution	[174]
	PyNHS/rGO/SPCE	anti-CRP	EIS	10 ng/mL	10 μg/mL–10 ng/mL	PBS buffer	[171]
PSA	GO/AuNPs composite	anti-total PSA and anti-free PSA	SWV and CV	total PSA: 0.2 ng/mL; free PSA: 0.07 ng/mL		PBS buffer	[175]
MDA	PARG-GQDs-GC	PARG	CV	0.329 nM	0.06–0.2 μM	PBS buffer and EBC	[176]
8-OHdG	ZnO NRs and ZnO NRs:rGO	anti-8-OHdG	CV	100 fg/mL	0.001–5.00 ng/mL	PBS buffer and spiked human urine	[177]
	ZnO NRs and ZnO NRs:rGO	anti-8-OHdG	CV	100 fg/mL	0.001–5.00 ng/mL	PBS buffer and spiked human urine	[177]
	C$u_{2}O$ SNPs@GOS/GCE label-free	-	CV	8.75 nM	0.02–1465 μM	Blood serum and urine	[178]
	GO–COOH/MWCNT–COOH/PEI/AuNP/GCE label-free	-	CV and DPV	7.06 nM	0.14–1.41 μM	Buffer and human urine	[179]
	D$y_{2}$ $O_{3}$-rGO/SPCE label-free	-	Amperometry	1.02 nM	0.05–135.3 μM	Human urine and blood serum	[186]
	MWCNT-rGO/GCE label-free	-	SWV	35 nM	3–75 μM	Buffer and human urine sample	[187]
	P-Arg/ErGO-AuNPs/GCE electrode label-free	-	DPV	1.0 nM	1–100 nM	Human urine sample	[182]
	ZnO-NFs/GOS/SPCE label-free	-	EIS	8.67nM	0.05–536.5 μM	Buffer and human urine sample	[188]
	ZnO/rGO/GCE/SPCE label-free	-	DPV	1.25 nM	5–5000 nM	Phosphate buffer and clinical human urine	[183]
Cyfra-21-1	ncCe$O_{2}$–rGO/ITO	anti-Cyfra-21-1	DPV	0.625 pg/mL	0.625 pg/mL–0.01 ng/mL	PBS buffer and spiked human saliva	[192]

Abbreviations: Graphene field-effect transistor (GFET); Graphene–Nafion field-effect transistor (GNFET); Pyrene-Tagged DNA Aptamer (PTDA); Electrochemical reduced graphene oxide (ERGO); Cardiac troponin I (cTnI); Glassy carbon electrode (ECL); Anoluminophore N-(4-aminobutyl)-N-ethylisoluminol functionalized graphene quantum dots (ABEI@GQDs); Ag–Ti$O_{2}$–reduced graphene oxide hybrid nanomaterial modified screen-printed electrode (Ag–Ti$O_{2}$–rGO/SPE); N-(4-aminobutyl)-N-ethylisoluminol functionalized graphene quantum dots (ABEI@GQDs); Microfilter platinum nanoparticles-decorated reduced graphene oxide (PtNPs-rGO); Screen-printed carbon electrode (SPCE); Poly arginine-graphene quantum dots (PARG-GQDs); Cytokeratin fradgment-21 (Cyfra-21-1); Cerium oxide nanocubes (ncCe$O_{2}$); Differential pulse voltammetry (DPV); monoclonal antibody (mAb); Indium tin oxide coated (ITO); ethanol Chemical Vapor Deposition treatment on top of pre-coated GO (ECVDGO); Quartz crystal microbalance (QCM); gold nanoparticles functionalized sulfur-doped graphene quantum dot (AuNPs/S-GQD); Hollow ZnS–CdS nanocage (h-ZnS-CdS NC); Square wave voltammetry (SWV); Nile blue (NB); Laser-scribed graphene electrode (LSG); Molecular imprinted polymer (MIP); Carbon paste electrode (CPE); Multiwalled carbon nanotubes (MWCNT); Dysprosium oxide nanoparticles (D$y_{2}$$O_{3}$); Procalcitonin (PCT); Aptamer carboxyethylsilanetriol (Apt-CES); Screen printed electrode (SPE); Polyethyleneimine (PEI); Atreptavidin (SA); 1,5-diaminonaphthalene (DN).

In optical biosensors, the biomolecule to be detected is fixed on an adequate substrate, which is interrogated with an electromagnetic field in the optical region. Frequently, molecular spectroscopy techniques, such as UV-VIS absorption/emission, infrared absorption, or Raman scattering are used for both the detection and quantification of the target analyte. The interaction of the optical field with biorecognition elements such as antibodies, enzymes, or nucleic acids, is the cornerstone on which the development of optical biosensors is based. The role of the GDMs substrate in optical biosensors is the modification of the analyte optical properties to improve its sensitivity, selectivity, or reliability detection. In subsequent paragraphs, we provide the principles and testing methods of optical biosensors most used for the detection of biomarkers.

#### 4.3.1. (A) Surface Plasmon Resonance Biosensors (SPR)

A SPR biosensor works based on the principle of surface electromagnetic evanescent waves between a metal dielectric interface and the change on its refractive index. This technique measures the refractive index change in the vicinity of thin metal layer (i.e., Au, Ag, or Al) in response to biomolecular interactions. Capturing agents are first immobilized on a metallic thin film and then a sample solution under study containing the analyte to detect is flowed across the SPR surface. The bindings of the analyte on the immobilized capture agent of the SPR metallic film causes changes on the refractive index on the biosensor [201], which can be measured as a shift of the SPR (the angle of minimum reflectivity). The SPR shift angle is directly proportional to the amount of the captured analyte. SPR technique is then especially suitable for the determination of interaction patterns between analytes (cells, proteins, nucleic acids), and antigens with the capturing agents (antibodies, enzymes, peptides, or DNAs) [202,203].

In order to enhance the sensitivity of the SPR biosensors, different nano structures attached to the metallic films have been tested. Graphene and GDMs, sometimes decorated with Au Nps, have been used for this purpose [203,204]. Enhancement factors of the SPR signal around 20-30 % were obtained when graphene or GDMs were sandwiched between the pristine metallic surface and the capturing agents. A considerable enhancement factor was obtained due to the strong excited electric field produced by the deposition of GDMs on the Au surface due to the processes of charge transfer between both materials.

#### 4.3.2. (B) Surface-Enhanced Raman Scattering (SERS) and Graphene-Enhanced Raman Scattering (GERS)

Raman scattering spectroscopy is a fast response and non-destructive analytical tool to identify and structurally characterize biomolecules. This technique also provides extensive information about the analyte conformational structure and induced changes due to microenvironment conditions [205,206]. Nevertheless, Raman spectroscopy has an intrinsic sensitivity lower than other molecular spectroscopies, which hinder its direct use for analytical detection and identification at low concentration (concentrations lower than 1 mM). To overcome this limitation, SERS has emerged as highly appreciated analytical strategy due to its capability to strongly enhance vibrational Raman modes of molecules when they are adsorbed on a metallic nanostructure (mainly Ag or Au) [195,207]. When molecules or biomolecules are close to the metallic surface their Raman bands can be enhanced by intensity factors ranging from 10${}^{2}$ to 10${}^{6}$, and even 10${}^{12}$, in the most favorable conditions. The SERS effect is based on the combination of the so-called electromagnetic and chemical mechanisms. The electromagnetic mechanism (EM) is based on the enhancement of the effective local electromagnetic field acting on a molecule when it is closely located to metallic NPs. Under these conditions, NPs act as nano-antennas by increasing the effective EM field, which is experienced by the molecules attached to them, resulting in the enhancement of their Raman scattering cross-section. On the other hand, the chemical mechanism (CM) is based on a charge transfer process between the NPs and the adsorbed molecules. The CM is a short-range effect that occurs on the molecular scale when the molecule is in contact, or very close, with the metallic NPs [205]. Despite the impressive enhancement factor obtained with SERS scattering, this approach commonly leads to spectral deformation, lowering the spectral quality (change of spectral profile, band broadening, and loss of resolution) that compromise the reliable identification of the analyte. As an alternative Raman scattering enhancement strategy the so-called GERS effect must be considered when the spectral deformation of SERS becomes a serious inconvenience for the identification of molecules. The Raman intensity enhancement by the absorption on GDMs is related with an increase in the molecule polarizability due to the sum of different effects, such as the high electronic density of graphenic substrates and energy/charge transfer process between the substrate and the adsorbed molecule. In general GERS is lower than SERS signal enhancement, but in some cases the combination of GERS and Drop Coating Deposition Raman (DCDR) methods, allowing enhancement factors similar to those of SERS [195]. Moreover, a promising approach is the use of GDMs substrates decorated with Au NPs, which combine the high enhancement factor and reduce the spectral profile disturbance of the attached molecule.

#### 4.3.3. (C) Fluorescence Resonance Energy Transfer (FRET)

FRET is a powerful analytical technique for target detection due to its sensitivity, simplicity, and reproducibility [208]. GDMs has been demonstrated as efficient quenchers for a variety of fluorophores, via non-radioactive electronic excitation energy transfer, and its large adsorption cross-section. GDMs offers a chemically susceptible 2D planar surface to incorporate donors such as quantum dots, NPs, biomolecules, and organic probes. The strategy in these applications relies on the high energy/electron transfer capability and the amphiphilicity of materials such as GO. These properties make GDMs capable of strong binding with biomolecules through π-π stacking and/or hydrogen bonding interactions, and at the same time quenching the fluorescence of nearby fluorescent dyes, by the process of energy transfer from the excited state of the dye to GDM. GDMs also have a tunable photo-luminescence property that arises from the small sp${}^{2}$ domains embedded in the sp${}^{3}$ matrix. Theoretical and experimental studies have indicated that both energy transfer and electron transfer can induce the quenching of fluorophores on graphene, while the quenched fluorescence could be recovered gradually with the increase in their distance, which offers a new idea for the development of fluorescent sensors [209].

#### 4.3.4. (D) Chemiluminescence Resonance Energy Transfer (CRET)

In contrast to FRET, CRET occurs via the specific oxidation of a luminescent substrate during chemiluminescence reaction without an external excitation source, thus, it can avoid the FRET drawbacks such as the requirement of simultaneous external excitation of both donor and acceptor fluorophores. CRET is a nonradiative dipole-dipole transfer of energy from a chemiluminescent donor to a suitable acceptor molecule without an external excitation source and it has been applied to detect various biological analytes.

### 4.4. Design and Fabrication of Optical Biosensors Based on GDMs

The developed methods for optical biosensing using GDMs, can be grouped in label-free and label-based strategies [210]. In the first group, the interaction of the material with the transducer generates a detectable spectroscopic signal. In the second group, the optical signal is generated through a labeled molecule that is identified by a colorimetric, fluorescent, or luminescent method. The label-free identification of biomarkers can also be achieved with Raman spectroscopy techniques such as GERS, which implies the use of a monolayer of GO or rGO over a reflective metallic substrate (Al or Si), where a fluorescent analyte is adsorbed and identified through its quenching of the fluorescence and the enhancement of its vibrational Raman modes [195,211]. On the other hand, in the label-based strategy, optical biosensors with GDMs have been designed by the synthesis of optical upconversion nanoparticles (UCNPs) exhibiting a FRET transfer signal to surface GO, allowing the sensitive and selective detection of viruses and antibodies [212]. A common strategy in fluorescence biosensors is the assembly of protein-specific fluorescent aptamers on the surface of GO or rGO nanosheets; for example, for sensitive detection of target IFN-γ biomarker in biological samples [196]. Recently, an interesting approach in optical biosensor was advanced by integrating a SPR optical fiber, coated with GO and silver and immobilized with antibodies, allowing the sensitive detection of human IgG [193].

#### Applications of Optical Biosensors in ID biomarkers

As in the case of electrochemical sensors, the use of GDMs enhanced optical biosensors has been mainly focused on cytokine detection. For example, de la O and coworkers explored the label-free detection of human IL-6 with Raman spectroscopy and through the deposition of a few layers of rGO over a Si support. The authors took advantage of the combination of DCDR and the GERS effects to detect IL-6, reaching a LOD lower than 1 pg/mL in PBS [195]. In addition, Lim and coworkers detected IL-5 in a label-free GO-based immunoassay, by taking advantage of the intrinsic fluorescence quenching of GO and through the induced peroxidase-catalyzed polymerization of DAB in a sandwich assay; the LOD reached was 5 pg/mL in PBS and 10 pg/mL in human serum [200] and the specificity for IL-5 was evaluated among other cytokines, such as IL-2, IL-17, IL-6, and IFN-γ. In addition, Yang and colleagues developed a CL immunosensor by immobilizing monoclonal ChIL-4 on a nitrogen-doped graphene-chitosan matrix, that allowed to detect chicken IL-4 in a LOD of 0.02 ng/mL in chicken serum samples, and high specificity was determined by adding analogous ChIFN-γ [199].

**Figure 3:**
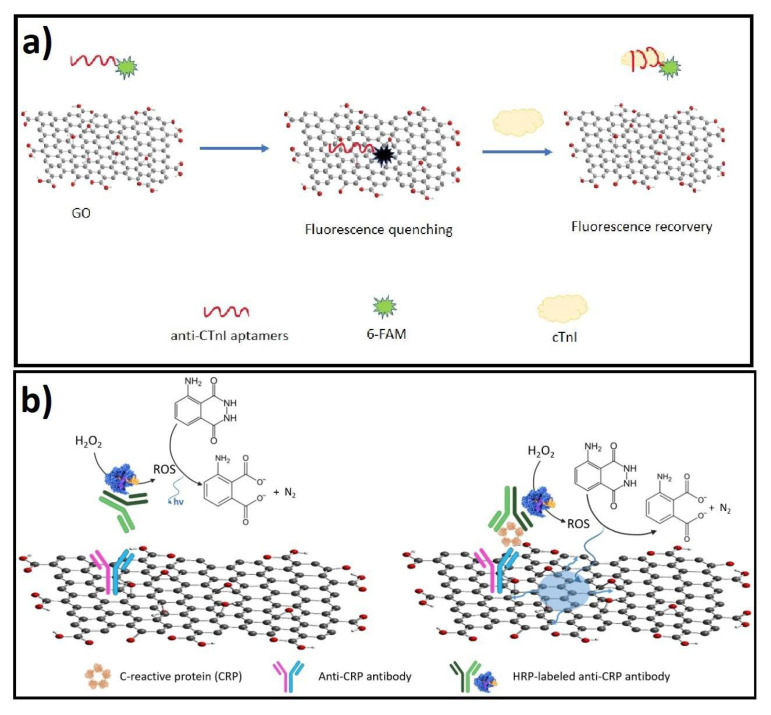
Optical Biosensors. (**a**) Fluorescence aptasensor based on a GO for fluorescence quenching and detection of cTnI. (**b**) Design and application of a CRET-type platform to detect CRP protein.

Additionally, IFN-γ was optically detected through fluorescence spectroscopy, by employing a double-stranded, dual-anchored, fluorescent aptamer on rGO nanosheets. This approach prevents non-specific quenching of fluorescent aptamer upon interaction with rGO, through hybridizing the fluorescent aptamer with a complementary sequence to form double strands [196]. The LOD of IFN-γ was 0.1 ng/mL with a total analysis time lower than 10 min, without the interference of IL-2 and TNF-α, used as non-target proteins. Using a similar fluorescence approach Liu and colleagues developed a fluorescence aptasensor based on a GO platform that led to the fluorescence quenching of the fluorescent anti-cTnI aptamer after binding with GO, which allowed researchers to detect the cTnI protein by reaching a LOD of 0.07 ng/mL in spiked serum samples [197]; specificity was evaluated by including several interference protein, such as HSA, BSA, IgA, and IgB (see Scheme in Figure 3a). On the other hand, BNP peptide was detected through a replicable FRET platform by employing GO as the FRET receptor, evaluating its on and off state on the presence of BNP in spiked blood and evaluating the performance in clinical samples, reaching a LOD of 45 fg/mL; no fluorescence restoration was obtained after replacing BNP with other competitive substances, such as IFN-γ, Cys, Arg, Gly, His, or HSA [198].

In addition, Lee and coworkers [194] designed a graphene-based CRET platform for homogeneous immunoassay of CRP detection through sandwich-type immunoreactions. The system enabled the capture of CRP at the concentration above 1.6 ng/mL in human serum samples, in the range observed during acute inflammatory stress. In this design graphene played a key role behaving as a more efficient energy acceptor than GO, while luminol served as a donor to graphene, triggering the CRET phenomenon (see Scheme in Figure 3b).

### 4.5. Wearable-Remote-Portable Biosensors

Compact, portable, and wearable devices in biosensor technology are attributes highly desirable for the simple, fast, sensitive, and continuous monitoring of physiological biomarkers in clinical patients [213]. Moreover, conventional detection methods such as immunoassays or liquid chromatography require several steps for sample preparation, operation, and analysis in prominent instruments; therefore, the miniaturization of sensor components into portable devices would significantly improve the biomarker POC [214]. GDMs within nano-micrometer size scale are ideal substrates for constructing miniaturized biosensors due to their surface area in nanostructured dimensions, and preserved physicochemical properties. Moreover, their excellent biocompatibility easily allows surface functionalization for anchoring a variety of target biomolecules, enabling the development of a wide variety of biosensors [215]. In addition, combining GDMs with biocompatible polymers has allowed the development of flexible and deformable sensor substrates that can be assembled into human wearable devices. In this section, we cover the novel development of remote, smart, wearable, and portable biosensor devices for the detection of inflammatory disease biomarkers.

GDMs are excellent materials for the design and fabrication of remote, miniaturized, wearable, and portable biosensors for biomarkers of inflammatory diseases. Remote biosensors are electronic devices that include the recognition of a biological component in the sensor, and the signal is recorded through a portable or smart device [216,217]. In those devices, the incorporation of graphene or GO layers, as conductive materials, allows researchers to perform the FET phenomenon, as the main sensing method for molecular target recognition [218]. Moreover, some wearable devices have been fabricated with diverse polymeric materials, allowing deformable and flexible biosensor qualities. For example, biocompatible and ultrathin polymeric films coated with graphene have allowed researchers to fabricate FET biosensors for the detection of cytokine biomarkers, such as IFN-γ and TNF-α [129,219]. Other strategies for developing cytokine biosensors have been improved by combining graphene monolayer-based FET-like structures with PDMS in a flexible substrate, where aptamers were immobilized and conductance measurements were performed in order to detect IFN-γ [220].

Designs of flexible and regenerative aptameric FET biosensors can be achieved by incorporating a graphene-Nafion composite film as a conducting channel between two Au electrodes. This composite film can be functionalized with aptamers for specific biorecognition of IFN-γ in the device [221]. In addition, robust, flexible supports for biosensors have been recently fabricated with printed technologies, such as the aerosol jet printing (AJP) technique, which contains graphene ink printed over polymeric sheets, that are further functionalized with antibodies for specific cytokine recognition [134]. Remote and wearable biosensors based on GDMs represent a promising field for developing robust, simple, and portable biosensing technology.

#### Applications of Portable-Remote-Wearable Biosensors in ID Biomarkers

IL-6 is the cytokine that has received the most attention in developing wearable devices, either alone or in multiple detection biomarkers. Hao and coworkers developed, for the first time, an integrated graphene-based portable system for wireless and online detection of cytokines, by developing an aptameric GFET and online signal-processing circuits, to wireless transmit the signal of the cytokine detection to a smartphone or external server through Wi-Fi connection. This portable system allowed the detection of IL-6 within a LOD of 10.5 pM in a buffer solution and 12.2 pM in human saliva, with the advantage of being portable and non-invasive [222]; the specificity was evaluated by including growth hormone and EGF in the solution; however, fluctuations were found to identify IL-6 in real saliva samples, due to the individual-to-individual uniformity.

**Figure 4:**
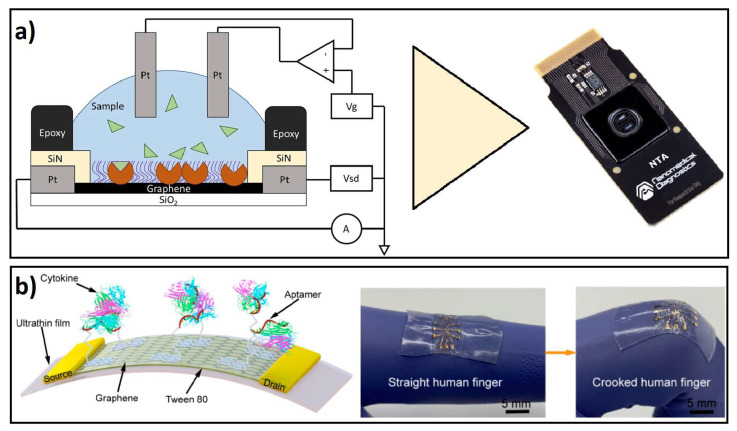
Wearable-remote-portable biosensors. (**a**) Design, manufacture, and parts of a FEB biosensor and its integration into a compact biosensor. (**b**) Design and manufacture of a flexible FET support for skin biosensor. *Source:* Figure adapted from (**a**) [224] and (**b**) [129] with permission of Springer and MDPI.

In addition, Afsahi and colleagues modified the FET effect biosensing for rapid biological testing of IL-6 to develop an integrated on-chip biosensor based on graphene. The sensing principle was based on the field-effect biosensing (FEB) technology, which measures the channel current and gate capacitance of an FET device containing immobilized biomolecular targets. This design was further integrated into a commercial AGILE R100 reader for measuring a change in conductance, allowing the sensitive detection of IL-6 only in PBS buffer with a LOD of 0.1 pM [223], showing improved performance in sensitivity, in comparison to commonly used assays; however, performance was not evaluated in biological samples. Following the same FEB principle, Goldsmith and coworkers improved and expanded the graphene biosensor technology, for the general opening of this technology, with an industrially manufactured on-chip graphene device that led to the fast and sensitive detection of IL-6 in an AGILE R100 reader, reaching a LOD of 2 pg/mL in PBS buffer solution. In spite that this device was not tested with biological or complex samples, it nicely shows the development of the next biosensor generation with low power requirements, compact and straightforward manufacturing [224] (Scheme in Figure 4a).

The single detection of TNF-α and IFN-γ has been detected with several graphene-based FET developments. For example, Hao and coworkers presented a substrate of SiO${}_{2}$ coated with polymer polyethene naphthalate (PEN), showing excellent flexible properties for wearable sensing applications of TNF-α in biological fluids, allowing its detection in PBS buffer by reaching a LOD of 26 pM and its selectivity was tested by including analogues IL-002 and IFN-γ into the biochemical functionalized graphene channel. [225]. Further, Farid and colleagues developed a solid GFET biosensor with specific DNA aptameric recognition of IFN-γ within a LOD of 83 pM in PBS solution. Furthermore, Wang and colleagues developed ultra-flexible and stretchable FET nano sensors based on a Mylar monolayer with aptameric immobilization on monolayer graphene, which allowed the separate detection of two different biomarkers: TNF-α in PBS buffer solution with a LOD of 5 pM [219], and IFN-γ in undiluted human sweat with a LOD down to 740 fM [221]; showing no interference of additional control biomarkers such as TNF-α, IL-002 or IL-6. Those developments showed further potential wearable applications for on-site monitoring of biomarkers on skin or tissue surfaces.

Moreover, the simultaneous detection of IFN-γ and TNF-α was shown by Wang and coworkers, through a deformable ultrathin substrate graphene-based FET biosensor, with wearable properties for time-resolved detection of cytokines in buffer solution and artificial tears, leading to LODs down to 2.75 and 2.89 pM for TNF-α and IFN-γ, respectively [129]. The device showed high specificity to IFN-γ and TNF-α even in the presence of related proteins such as EGF and GH and time-resolved measurements were shown for TNF-α (see scheme in Figure 4b).

On the other hand, a recent report by Hao and colleagues showed the development of a robust aptameric dual-channel graphene-FET device with flexible wearable properties, allowing the simultaneous and remote measurement, through a smartphone, of targeting inflammatory cytokines involved in the progression to severe or critical COVID-19 cases. This novel device allowed the independent detection of IFN-γ, TNF-α, and IL-6 with LODs of 476, 608, and 611 fM, respectively, in diverse biofluids, including serum, saliva, urine, and sweat; showing accurately and rapid detection (around 7 min), that can improve to monitor conditions of COVID-19 in patients [226].

Furthermore, a graphene-based optical biosensor was presented for detection of ChIFN-γ assisted with a flow-through CL immunoassay, where GO nanosheets were introduced into a CL immunoassay for capture antibody immobilization, allowing a LOD of 0.36 pg/mL in PBS solution showing good specificity, stability, and fabrication reproducibility [227].

The sensitive detection of CRP is desirable for an accurate diagnosis of SARS-CoV-2. In that sense Torrente and colleagues showed the development of a wireless graphene-based multiplexed, portable, and electrochemical platform that allowed the multiple detections, through amperometric measurements, of CRP, IgM, and IgG in human saliva and positive patient serum samples, allowing a LOD of 250 ng/mL of CRP [228]; high specific binding for SARS-CoV-2 was observed in comparison with biomarkers of related coronaviruses, such as SARS-CoV and MERS-CoV.

Moreover, Vashist and colleagues developed an optical biosensor for CRP detection through a graphene-based immunoassay coupled to a smartphone-based colorimetric reader that led to CRP detection in clinical and diluted human whole blood samples, reaching a LOD of 0.07 ng/mL, without the interference of common proteins such as LCN2, HFA, HSA, IL-1β, IL-6, IL-8, and TNF-α [229]. A detailed description of relevant wearable-remote-portable biosensors assisted with GDMs is presented in Table 4.

## 5. Discussion

Biosensors based on graphene and its derivatives have shown potential benefits in detecting biomarkers due to their significant advantages, including their excellent conductivity, adjusting to electrical, electrochemical, and optical readings, and binding specific ligands that can functionalize their surfaces. GDMs production needs to be improved to obtain graphene, GO, or rGO flakes with reproducible physicochemical properties through a strict surface size control.

In the last two decades, the development of highly effective biosensors has achieved selective, sensitive, accurate, reproducible, stable systems, and data can be obtained in minutes; this leads to biomarkers being detected in biological samples in the order of picomolar (pM). It seems clear then that the increasing use of GDMs will result in significant progress in developing sensors and other health devices in the following years. According to its operating principles, electrochemical and optical biosensors are the most widely investigated graphene-based biosensors.

Electrochemical biosensors have high sensitivity, detection capacity, and selectivity. These types of biosensors lack a wide range of surface architectures that allow increased sensitivity for identifying the electrochemical fingerprint in response to a biochemical event. Because molecular interaction results from electrostatic interactions, ionic charge changes in the environment affect the biosensor response; then, as a solution to this problem, the dimensions of the sensor have been reduced to increase the signal and in conjunction with a biomolecule to direct the specificity of the biosensor in each event. In the field of voltammetric biosensors, devices with a sensitivity of femtomolar have been developed, becoming a tool for rapid diagnosis for point-of-care applications when combined with DNA or molecule to detect biomarkers. In the case of impedance, biosensors that use labels are highly selective and sensitive; however, their preparation has an expensive cost, which has led to the guidelines to increase research directed at the modification of electrodes with nanomaterials to increase detection sensitivity. Similar to the electrochemical biosensors mentioned in this review, amperometry biosensors use DNA, enzymes, or other biomolecules as ligands, increasing specificity. It can have a direct or indirect signal in this type of biosensor. It is a low-cost technology and the sensing is proportional to the concentration of electroactive species, which has allowed it to become one of the most-used techniques for designing electrochemical biosensors. In recent years, electrochemical biosensors have had a significant impact since they have been used to detect viruses, such as SARS-CoV2, bacteria as Mycobacterium tuberculosis, among other pathogens, with a femtomolar sensitivity.

Optical biosensors have a significant impact on medicine, biotechnology, and the environment. Based on the ligand optical property in response to the analyte interaction, most optical biosensors analyzed in the literature are biosensors based on fluorescence, chemiluminescence, SERS, and plasmon. Optical biosensors are analytical devices as they contain an integrated element to increase biorecognition in conjunction with the detection technique used. These elements can be biological materials, polypeptides of various natures, and tissues.

SPR-based biosensors can contribute to the analysis of kinetic events, equilibrium, and concentration in real-time without additional labeling. Sensing with SERS results in a fast and sensitive method, one of the significant advantages of this technique is the detection capacity in biological samples. It also allows for the detection of a single molecule by amplifying the intensity of the Raman signal. Fluorescence-based biosensors characterize the intrinsic fluorescence of various biomolecules with a ligand or when the ligand binds to the biomolecule; however, molecular interaction can be detected by labeling the ligand with a component capable of fluorescing. An alternative to increase sensitivity is based on association with nanostructures in general and GDMs, which are becoming increasingly important in detecting this biomarker. On the other hand, labels in the biosensor can alter sensing due to conformational changes, steric hindrance, and uncontrolled orientation. To counteract these effects, there are label-free biosensors; however, this alternative reduces the sensitivity and specificity of the device.

Optical sensors combined with smart, remote, and wireless pathways present competitive advantages for rapid disease diagnosis by providing real-time reading and exceptional qualities such as high detection limits, specificity, biocompatibility, and sensitivity. They also enable portable, fast, and affordable instrumentation that can be further miniaturized into practical POCs.

Biomarker detection has led to the development and improvement of biosensors for sensing various types of body fluids. Devices with this approach must be precise, the interaction of the ligand and the analyte must be independent of the physical and chemical parameters used in each technique, and the response must be exact, reproducible, and non-invasive. Saliva has been explored to detect biomarkers, bacteria, and viruses in this sense. Electrochemical biosensors have demonstrated a sensitivity of the order of picograms in detecting some biomarkers of oral cancer, such as interleukins and enzymes. In the case of optical biomarkers and biomarkers, bacteria and viruses have been detected in the case of saliva and blood biomarkers of various inflammatory diseases (meningitis, cancer, among others).

Furthermore, particular attention should be paid to the study of interactions between all components of the active element of biosensors to optimize their design and fabrication, combining novel spectroscopic techniques and computational methods to study the interaction forces between the components in the sensor and the attached biomolecule. These strategies might be relevant to modulating the molecular interaction forces to favor the specific and highly sensitive detection of a particular biomarker on the biosensor surface.

With this theoretical and experimental information, adequate physicochemical manipulation strategies could be defined to modulate the molecular interaction forces to favor the specific and high sensitivity of detecting a particular biomarker on the biosensor surface. The control of the type and amount of oxygen species present on the edge and the basal plane of the GO or rGO flakes can significantly modify the materials’ electrochemical and optical responses, becoming of primary importance to obtain the desired molecular interaction on the biosensor’s active surface.

Another relevant aspect that still needs to be advanced for an accurate clinical diagnosis is the development of robust devices to recognize multiple biomarkers simultaneously, which would allow the specific detection of one or several diseases in a single sensor. This capability will be critical for the study of complex biofluids, and in this regard, it is also essential to consider the use of additional less invasive, biological fluids such as saliva, sweat, or tears. In this sense, advanced selectivity performance must be reached to study complex or non-pretreated samples; therefore, implementing advanced microfluidic strategies in continuous sensing devices could significantly progress this field.

In addition, other relevant considerations to drive the progress of POC biosensors are related to the development of miniaturized portable biosensors for continuous and remote monitoring of biomarkers, allowing easy exchange and communication of data between patients and clinicians. Moreover, the development of new biosensor technology in terms of portability should benefit with low prices, quick detection, high sensitivity, possible reuse, and measuring biomarkers at a personal level; however, challenges still need to be overcome; for example, those devices need to be evaluated considering physiological parameters such as skin mechanics, deformation, wetting resistance. Further, in the specific case of wearable and implantable biosensors, it is highly relevant to update and further explore the non-toxicity of most GDMs. Furthermore, new materials and nanomaterials with GDMs should satisfy relevant, validated parameters such as selectivity, sensitivity, wide dynamic range, fast response, and reproducibility.

A clear example of the need to increase biosensor research and development efforts is to achieve the effective, versatile, and fast implementation of techniques for the early, rapid, and accurate detection of highly infectious viruses such as SARS-CoV-2, or the so-called superbugs, which would allow us to achieve timely diagnosis, therapy, and efficient action to prevent the spread of these microorganisms. This is of global relevance, considering the role of biosensors in the early and rapid detection of viral diseases, which in the future could help in critical pandemics.

Moreover, recent advances in nanotechnology and new materials provide alternatives to optimize the design of biosensors, leading to fast, accurate, and precise monitoring. In addition, nanoparticle-based materials contribute to increased sensitivity and specificity in electrochemical and optical biosensors, thus becoming an essential component in biosensors. The immobilization of analytes in nanomaterials is the key to increasing sensitivity and reducing detection limits. In the case of electrochemical biosensors, this contributes to real-time measurement with high specificity and amplifies the signal. This technology has been compared with conventional methods such as immunofluorescence, real-time PCR, and cell cultures; as a result, the electrochemical biosensors are considered a tool for detecting pathogens, biomolecules, and cells in the diagnosis of diseases and even in environmental monitoring. The optic-based biosensors are considered analytical devices for detecting molecular events within a cell; their efficiency in reproducibility and reliability depends on the immobilization of receptors in a targeted and controlled manner toward the maximum sensitivity and selectivity of the assay. Label-free biosensors are moving towards automation to detect and quantify various analytes in a biological sample in minutes with high sensitivity. The biosensors must be directed towards the industrialization of stable devices with immobilized receptors or ligands, maintaining their biological activity. A relatively new alternative is the generation of biosensors combining both the electrochemical and optical parts, proposing the generation of innovative devices for detecting biomarkers with very high sensitivity.

## 6. Conclusions

This review describes the potentialities, benefits, and recent advances of GDMs to develop biosensors for chronic inflammatory diseases biomarkers detection. We have highlighted the main physicochemical features of GDMs that make them especially attractive for developing high sensitivity biosensors, with a particular focus on electrochemical and optical platforms and devices for detecting inflammatory diseases biomarkers. Furthermore, we have explored the novel development and applications of GDM-based biosensors into smart, wearable, and portable devices that could optimize the clinical diagnosis of inflammatory diseases.

So far, in this review, we have shown the potentialities, benefits, and opportunities of graphene-based materials to improve health diagnosis through the development of novel biosensors that will have a relevant impact on life sciences and that will open new fields of applications. Special mention should be made of the importance of developing novel POC biosensors with a potential broad impact on the market, to the extent that they will allow, maintaining the reliability of the clinical data provided, to facilitate the timely diagnosis of the evolution of a medical condition, improving the fast and efficient communication between patients and clinicians.

## Figures and Tables

**Table 1:** Main molecules that constitute the chronic inflammatory disease as potential biomarkers.

Molecular Biomarker	Disease	Ref.
Pro-inflammatory cytokines, interleukins (IL): IL-1β , IL-6, IL-8, IL-15, IL-17, IL-18	Periodontitis, psoriasis, COVID-19, rheumatoid arthritis, oral cancer, Alzheimer’s disease, osteoarthritis.	[41,62,63,64,65]
Anti-inflammatory cytokines: IL-4, IL-10, IL-13	Asthma, inflammatory pain, ulcerative colitis, systemic sclerosis.	[66,67,68,69]
Others cytokines: IL-3; IL-5, IL-12	Primary open angle glaucoma, inflammatory bowel disease, asthma	[70,71,72,73,74]
serum cardiac troponin I (cTnI)	Cardiac troponin T, coronary artery disease	[75,76,77]
B-type natriuretic peptide (BNP)	Coronary artery disease	[78]
Pro-inflammatory cytokine tumor necrosis factor-alpha (TNF-α)	Rheumatoid arthritis, immune reconstitution inflammatory syndrome, inflammatory bowel disease	[79,80,81]
Monocyte chemoattractant protein-1 (MCP-1)	Alzhemier’s disease, periodontal disease	[82,83]
Interferons (IFNs)	Rheumatoid arthritis, associated vasculitis, Alzheimer’s disease, inflammatory bowel disease, severe acute respiratory syndrome	[84,85,86,87,88]
High-mobility group box 1 (HMGB1)	Atherosclerosis, COVID-19, influenza	[89,90,91]
Enzymatic anti-oxidants superoxide dismutase (SOD), glutathione peroxidase (GPx), NADPH oxidase2 (NOX2), inducible nitric oxide synthase (iNOS), cyclooxigenase, (COX-2)	Tuberculosis, Parkinson, metabolic syndrome, lupus erithematosus, periodontitis, neurodegenerative disease, colitis, virus-associated human malignant neoplasm	[92,93,94,95,96,97,98]
Malondialdehyde (MDA)	Diabetes mellitus, psoriasis	[99,100]
8-Hydroxy-2′-deoxyguanosine (8-OHdG)	Chronic obstructive pulmonary disease, colorectal cancer	[101,102]
C-reactive protein (CRP)	Cardiovascular disease, COVID-19, bacterial infection	[103,104]
Transforming growth factors (TGFs)	Glomerulonephritis, angiogenesis	[105,106]
High-mobility group box 1 (HMGB1)	COVID-19, oral inflammation	[90,104]
Reactive oxygen species (ROS)	Chronic inflammatory joint	[107]

**Table 3:** Summary of optical biosensors assisted with GDMs for detection of inflammatory disease biomarkers.

Biomarker	Sensing Platform Design	Surface Modification	Detection Method	LOD	Detection Range	Type of Sample	Ref.
IgG	Ag-GO bilayer film	Goat anti-human IgG	SPR	0.04 μg/mL	5–30 μg/mL	PBS buffer	[193]
CRP	graphene-based CRET platform	anti-CRP	CL	0.93 ng/mL	1–1000 ng/mL	PBS buffer and human serum	[194]
IL-6	rGO/Si	label-free	GERS	1 pg/mL	10 μg/mL–1 pg/mL	PBS buffer	[195]
IFN-γ	DAGO	dsDA-Aptamer, label	Fluorescence	0.1 ng/mL	100 pg/mL–10 μg/mL	Buffer and spiked human serum	[196]
cTnI	FMAA-GO	anti-cTnI aptamer	Fluorescence	0.07 ng/mL	0.10–6.0 ng/mL	Tris–HCl buffer and spiked human serum	[197]
BNP	GO nanosheet	FAM-aptamer	FRET	45 fg/mL	0.074–0.56 pg/mL	Buffer, spiked blood and clinical blood	[198]
IL-4 (chicken)	(NG)-chitosan nanocomposite	mAb ChIL-4	flow-through CL	0.02 ng/mL	0.05–70 ng/mL	PBS buffer and spiked in chicken serum	[199]
IL-5	GO sheets on amine-modified glass surface	DAB/anti-IL-5 and HRP-anti-IL-5	Fluorescence Quenching	5 pg/mL in PBS; 10 pg/mL in human serum	0.005–0.5 ng/mL	Phosphate buffer and spiked human serum	[200]

Abbreviations: Surface plasmon resonance (SPR); Fluorescence resonance energy transfer (FRET); Graphene Enhanced Raman Spectroscopy (GERS); Fluorophore carboxyfluorescein (FAM); Cardiac troponin I (cTnI); B-type natriuretic peptide (BNP); 5′-6-FAM-modified anti-cTnI aptamers (FMAA); Double-stranded and dual- anchored fluorescent aptamer on rGO nanosheets (DAGO); Chemiluminescent (CL); Chicken interleukin-4 (ChIL-4); Nitrogen-doped graphene (NG); Horseradish peroxidase (HRP).

**Table 4:** Summary of wearable-remote-portable biosensors assisted with GDMs for detection of inflammatory disease biomarkers.

Biomarker	Sensing Platform Design	Surface Modification	Detection Method	LOD	Detection Range	Type of Sample	Ref.
Il-6	miniaturized GFET nanosensing system	IL-6-specific-aptamer	Aptameric GFET	10.5 pM (testing solution), 12.2 pM (saliva)	10–100 nM	PBS buffer, gargle solution and human saliva	[222]
	Foundry-Fabricated Graphene Sensors	anti IL-6	graphene-enabled FEB	<2 pg/mL	2–1000 pg/mL	PBS buffer solution	[224]
	Graphene chip integrated in AGILE R100	Anti-IL-6	FEB with AGILE R100 reader	0.1 pM	0.01–10 pM	PBS buffer	[223]
TNF-α	flexible graphene aptameric nanosensor	aptameric DNA	GFET nanosensor	26 pM	50 pM–500 nM	PBS buffer	[225]
	ultraflexible GFET nanosensor device	DNA aptamer	FET	5 pM	50–100 pM	PBS buffer	[219]
TNF-α and IFN-γ	Wearable and deformable GFET	Aptamers	GFET	TNF-α: 2.75 pM; IFN-γ: 2.89 pM	-	PBS buffer and spiked artificial tears	[129]
IFN-γ (single)	Liquid gate FET-like transistors	IFN-γ aptamer	FET	83 pM	0 nM–100 μM	PBS buffer	[220]
IFN-γ and ChIFN-γ	flexible and regenerative aptameric GNFET	IFN-γ aptamer	FET	740 fM	0.015–250 nM	PBS buffer and spiked in human sweat	[221]
	flow-through CL immunoassay	Anti-ChIFN-y	CL coupled to flow-through	0.36 pg/mL	0.001–0.1 ng/mL	PBS buffer, supernatant and infected serum samples	[227]
CRP	Graphene immunoassay with smartphone-based reader	anti-human CRP	Smartphone-based colorimetric reader	0.07 ng/mL	0.03–81 ng/mL	clinical and diluted human whole blood	[229]

Abbreviations: Surface plasmon resonance (SPR); Fluorescence resonance energy transfer (FRET); Graphene Enhanced Raman Spectroscopy (GERS); Fluorophore carboxyfluorescein (FAM); Cardiac troponin I (cTnI); B-type natriuretic peptide (BNP); 5′-6-FAM-modified anti-cTnI aptamers (FMAA); Double-stranded and dual- anchored fluorescent aptamer on rGO nanosheets (DAGO); Chemiluminescent (CL); Chicken interleukin-4 (ChIL-4); Nitrogen-doped graphene (NG); Horseradish peroxidase (HRP).

## Data Availability

Not applicable.
